# Additively Manufactured Open-Cell Porous Biomaterials Made from Six Different Space-Filling Unit Cells: The Mechanical and Morphological Properties

**DOI:** 10.3390/ma8041871

**Published:** 2015-04-21

**Authors:** Seyed Mohammad Ahmadi, Saber Amin Yavari, Ruebn Wauthle, Behdad Pouran, Jan Schrooten, Harrie Weinans, Amir A. Zadpoor

**Affiliations:** 1 Faculty of Mechanical, Maritime and Materials Engineering, Delft University of Technology (TU Delft), Mekelweg 2, 2628 CD Delft, The Netherlands; E-Mails: s.aminyavari@tudelft.nl (S.A.Y.); b.pouran@tudelft.nl (B.P.); h.h.weinans@tudelft.nl (H.W.); a.a.zadpoor@tudelft.nl (A.A.Z.); 2 LayerWise NV, Kapeldreef 60, 3001 Leuven, Belgium; E-Mail: ruben.wauthle@layerwise.com; 3 Department of Orthopedics and Department of Rheumatology, University Medical Center Utrecht, Heidelberglaan 100, 3584 CX Utrecht, The Netherlands; 4 Department of Metallurgy and Materials Engineering, KU Leuven, Kasteelpark Arenberg 44, PB 2450, 3001 Leuven, Belgium; E-Mail: Jan.Schrooten@mtm.kuleuven.be

**Keywords:** cellular solids, selective laser melting, compressive properties, and porous Ti alloy

## Abstract

It is known that the mechanical properties of bone-mimicking porous biomaterials are a function of the morphological properties of the porous structure, including the configuration and size of the repeating unit cell from which they are made. However, the literature on this topic is limited, primarily because of the challenge in fabricating porous biomaterials with arbitrarily complex morphological designs. In the present work, we studied the relationship between relative density (RD) of porous Ti6Al4V EFI alloy and five compressive properties of the material, namely elastic gradient or modulus (E_s20–70_), first maximum stress, plateau stress, yield stress, and energy absorption. Porous structures with different RD and six different unit cell configurations (cubic (C), diamond (D), truncated cube (TC), truncated cuboctahedron (TCO), rhombic dodecahedron (RD), and rhombicuboctahedron (RCO)) were fabricated using selective laser melting. Each of the compressive properties increased with increase in RD, the relationship being of a power law type. Clear trends were seen in the influence of unit cell configuration and porosity on each of the compressive properties. For example, in terms of E_s20__–70_, the structures may be divided into two groups: those that are stiff (comprising those made using C, TC, TCO, and RCO unit cell) and those that are compliant (comprising those made using D and RD unit cell).

## 1. Introduction

In orthopaedic surgery, cellular structures are used as three-dimensional porous biomaterials that try to mimic the structure and function of bone [1]. The porous biomaterial could be used either as a bone substitute or a cell-seeded scaffold used as a part of a tissue engineering approach. In either case, the porous biomaterial should be designed such that its mechanical properties match those of bone, while considering the other factors that maximize bone ingrowth. For example, the permeability of the porous structures used in bone tissue engineering could influence cell migration and mass transport and should be carefully designed [2,3]. During the last two decades, several design principles have been proposed for the design of bone tissue engineering scaffolds that consider the mechanical properties, biocompatibility, biodegradability, and bio-functionality of the scaffold biomaterials [4,5,6,7,8,9].

In this study, we focused on the compressive properties of porous titanium biomaterials aimed for application in orthopaedic surgery. Solid titanium alloys are often very stiff, exceeding the mechanical properties of bone by up to one order of magnitude [10,11]. The mismatch between the mechanical properties of bone and those of the biomaterial could hinder bone ingrowth, result in stress shielding, bone resorption, and ultimately cause loosening of orthopaedic implants [12,13,14,15]. Creating porous structures out of bulk materials, however, results in much lower stiffness values that are comparable with those of bone [10,16,17]. Traditionally, various techniques have been used for fabrication of porous biomaterials including space-holder method, hot isostatic pressing, gel casting, and chemical vapor deposition/infiltration [18,19,20,21]. Recently, additive manufacturing techniques have been introduced for manufacturing of porous biomaterials and have several advantages over conventional techniques including their ability to create arbitrarily complex 3D structures, highly accurate and predictable porous structure, and wide materials selection [22,23,24,25]. Two widely used AM methods are selective laser melting [26,27,28,29,30] and electron beam melting [31,32,33,34]. In addition to favorable mechanical properties, highly porous biomaterials have a large pore space that could be used for controlled release of growth factors [35] as well as huge surface area that could be treated using chemical and electrochemical techniques for obtaining desired bio-functional properties [36,37,38,39].

The mechanical properties of additively manufactured porous biomaterials are highly dependent on the type of unit cell from which they are made [40,41,42,43,44,45]. Optimizing the mechanical properties of porous biomaterials for different applications may require combining various types and dimensions of unit cells in one single porous structure. It is therefore important to have a good understanding of the relationship between the type and dimensions of unit cell and the resulting mechanical properties of the porous structure [46]. Many different types of unit cells are available. However, data on the mechanical properties of porous structures from many different unit cell configurations are limited.

In the present work, we used six different unit cell configurations, namely, cubic, diamond, truncated cube, truncated cuboctahedron, rhombic dodecahedron, and rhombicuboctahedron are considered in the current study. For each of these configurations, we used selective laser melting to manufacture porous structures with different porosities. Micro-CT imaging and compression testing were performed to determine the morphological and mechanical properties of the porous materials and to study the relationship between these parameters.

## 2. Materials and Methods

### 2.1. Materials and Manufacturing

**Figure 1:**
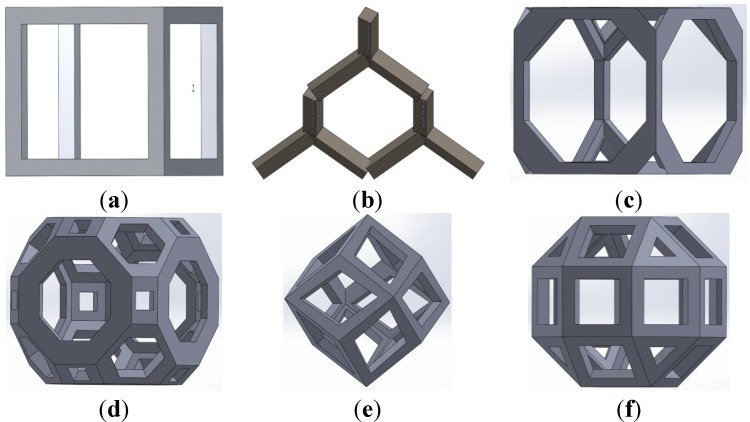
Schematic drawings of the unit cells used in the porous structure: (**a**) Cubic; (**b**) Diamond; (**c**) Truncated cube; (**d**) Truncated cuboctahedron; (**e**) Rhombic dodecahedron; (**f**) Rhombicuboctahedron.

**Figure 2:**
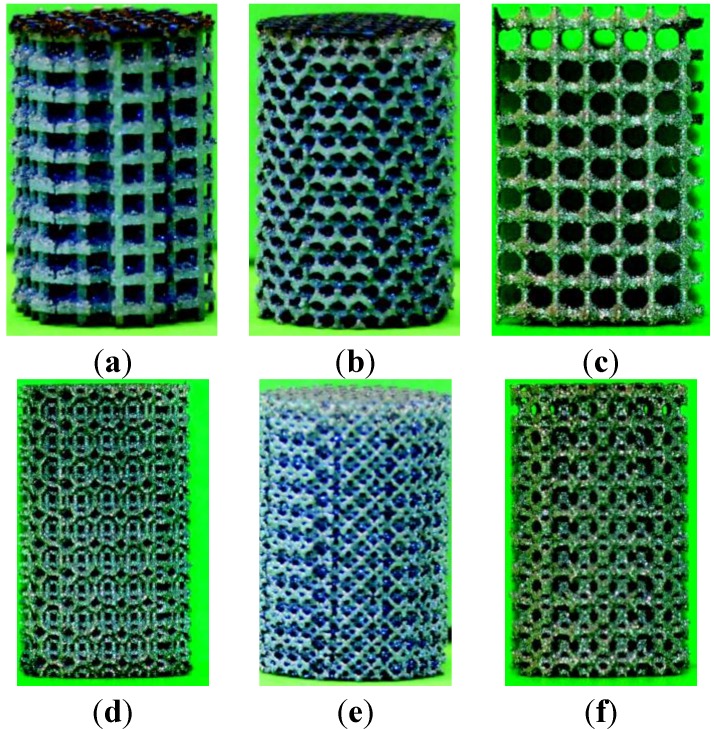
Sample specimens from the porous structures based on different types of unit cells: (**a**) Cubic; (**b**) Diamond; (**c**) Truncated cube; (**d**) Truncated cuboctahedron; (**e**) Rhombic dodecahedron; (**f**) Rhombicuboctahedron.

Selective laser melting (SLM) method (Layerwise NV, Leuven, Belgium) was used for processing of Ti6Al4V-ELI powder (according to ASTM B348, grade 23) on top of a solid titanium substrate and in an inert atmosphere. Porous titanium structures were thereby manufactured based on six different unit cells configurations, namely, cubic, diamond, truncated cube, rhombicuboctahedron, rhombic dodecahedron, and truncated cuboctahedron (Figure 1). The details of the laser process technique were reported in our previous studies [10,16,40,47,48]. For each unit cell, different porosities were achieved by changing the strut thickness and pore size (Table 1). Cylindrical specimens with the length of 15 mm, diameter of 10 mm and unit cell size of 1.5 mm were manufactured for static compression testing (Figure 2). After fabrication, electro discharge machining (EDM) was used to remove the specimens from the substrate.

**Table 1:** Morphological properties of the porous structures used.

	Strut Diameter (μm)		Pore Size (μm)	
	Nominal (Design)	μCT (SD)	Nominal (Design)	μCT (SD)
**Cubic (C)**				
C-1	348	451 (147)	1452	1413 (366)
C-2	540	654 (190)	1260	1139 (359)
C-3	612	693 (200)	1188	1155 (354)
C-4	720	823 (230)	1080	1020 (311)
**Diamond (D)**				
D-1	277	240 (46)	923	958 (144)
D-2	450	416 (65)	750	780 (141)
D-3	520	482 (70)	680	719 (130)
D-4	600	564 (76)	600	641 (137)
**Truncated Cube (TC)**				
TC-1	180	331 (76)	1720	1625 (398)
TC-2	240	363 (80)	1660	1615 (392)
TC-3	304	395 (88)	1596	1593 (382)
TC-4	380	463 (126)	1520	1535 (370)
TC-5	460	568 (183)	1440	1497 (360)
TC-6	530	620 (200)	1370	1426 (357)
**Truncated Cubeoctahedron (TCO)**				
TCO-1	324	350 (60)	876	862 (349)
TCO-2	460	416 (64)	1040	1142 (383)
TCO-3	520	452 (65)	980	1098 (386)
TCO-4	577	482 (70)	923	1079 (391)
TCO-5	621	516 (82)	862	1065 (361)
TCO-6	693	564 (76)	807	1049 (383)
**Rhombicdodecahdron (RD)**				
RD-1	250	246 (53)	1250	1299 (449)
RD-2	310	305 (97)	1190	1224 (455)
RD-3	370	440 (126)	1130	1168 (364)
RD-4	430	461 (163)	1070	1305 (554)
RD-5	490	430 (122)	1010	920 (300)
RD-6	550	506 (144)	950	1058 (356)
**Rhombic Cubeoctahedron (RCO)**				
RCO-1	380	348 (59)	820	877 (355)
RCO-2	410	369 (59)	790	847 (349)
RCO-3	440	486( 113)	760	1089 (402)
RCO-4	470	437 (61)	730	754 (359)
RCO-5	500	539 (120)	700	1043 (401)
RCO-6	530	438 (61)	670	794 (368)

### 2.2. Morphological Characterization

For morphological characterization, we scanned the titanium scaffolds using a micro-CT (Quantum FX, Perkin Elmer, Waltham, MA, USA). The scans were made under tube voltage of 90 kV, tube current of 180 μA, scan time of 3 min, and resolution of 42 μm. The 3D images of the porous structures were automatically reconstructed using the in-built software of the micro-CT. The reconstructed images were then transferred to the Caliper Analyze 11.0 (provided by the manufacturer) to align the geometry along the major axis of the specimens and to acquire 2D slices. The 2D slices contained transverse views of the scaffolds, *i.e.*, circular cross-sections. The 2D slices were then imported into the ImageJ 1.47v (http://imagej.nih.gov/ij/) in order to create region of interests (ROIs) and segment the titanium volume using the optimal thresholding algorithm available in the boneJ [49] plugin of ImageJ 1.47v (16 bit images). Segmented images were then exported to the boneJ plugin to calculate the ratio of the void volume to the 3D ROI volume that was ultimately reported as the structure relative density of the porous structures.

In addition, the Archimedes technique and dry weighing were used for determining the structure relative density of the specimens (Table 2) using five specimens from each porous type of porous structure, except for the case of rhombic dodecahedron unit cells that only 2 samples were used for measurement of the Archimedes porosity values. In both cases, an OHAUS Pioneer balance was used for weight measurements that were performed in normal atmospheric conditions in room temperature. As for the dry weighing, the weight of the porous specimens was divided by the theoretical weight of the corresponding solid specimens assuming a theoretical density of 4.42 g/cm^3^ for Ti6Al4V-ELI [50]. In the Archimedes technique, the specimens were weighed both in dry conditions and in pure ethanol to determine the actual and macro volume and calculating overall porosity of the porous structures.

### 2.3. Compressive Testing

The mechanical properties of the porous structures were obtained by static compression test using a static test machine (INSTRON 5985, 100 kN load cell) by applying a constant deformation rate of 1.8 mm/min. The compression test was carried out in accordance with ISO standard 13314:2011 [51] which refers to mechanical testing of porous and cellular metals. The tests were continued until 60% strain was applied to the specimens. Five specimens were tested for every variation of the porous structures. The stress-strain curves were obtained and the mean and standard deviation of each of five compressive properties were determined. According to the above-mentioned standard, the elastic gradient (Eσ_20–70_) was calculated as the gradient of the elastic straight line between two stress values, namely σ_70_ and σ_20_. The first maximum compressive strength (σ_max_) that corresponds to the first local maximum in the stress-strain curve was also calculated. The plateau stress (σ_y_) was defined according to the same standard as the arithmetical mean of the stresses between 20% and 40% compressive strain and was calculated for all specimens. [40,51]. Energy absorption, which is defined as the energy required for deforming a specimen to a strain (ε), was calculated from the area under the strain-stress curve up to 50% strain [52,53].

In order to analyze the compressive properties of porous structures more systematically, power laws relating structure relative density (the weight per unit volume of a material, including voids that exist in the tested material” as defined in ASTM D1895) to different compressive properties were fitted to the measured experimental data:

(1) $$X=a{\text{ρ}}^{b}$$

where *X* is any of the above-mentioned compressive properties measured for the porous structures and ρ is structure relative density. The parameters *a* and *b* are dependent on the type of the unit cell.

### 2.4. Correlational Analysis

MATLAB and Simulink R2014a, The MathWorks Inc., Natick, MA, USA, and Microsoft Excel, Microsoft Corporation, Redmond, WA, USA, were used to determine the correlation between the compressive properties of specimens and relevant density. Closeness of the data to the fitted regression line was measured by coefficient of determination.

## 3. Results

**Figure 3:**
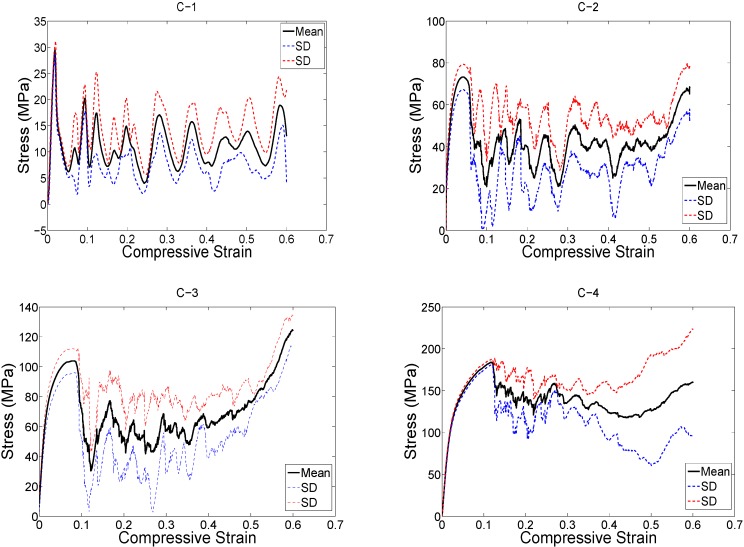
Compressive stress-*versus*-compressive strain curves for specimens based on the cube unit cell and with different porosities (see Table 2).

**Figure 4:**
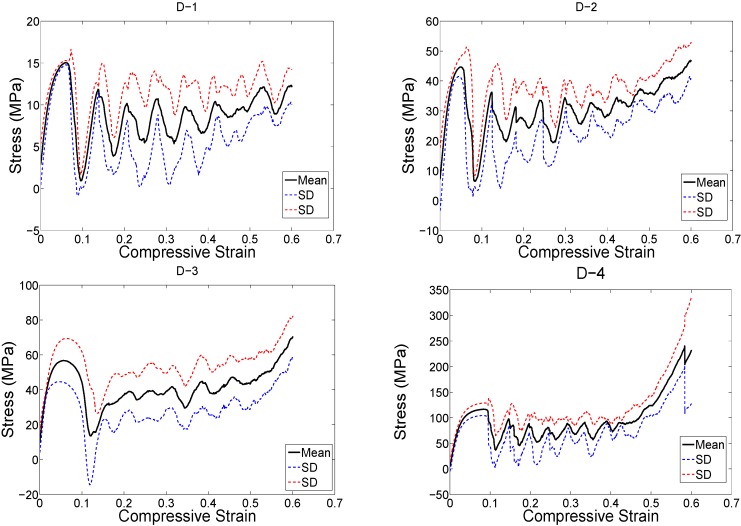
Stress-strain curves for specimens based on the diamond unit cell and with different porosities (see Table 2).

**Figure 5:**
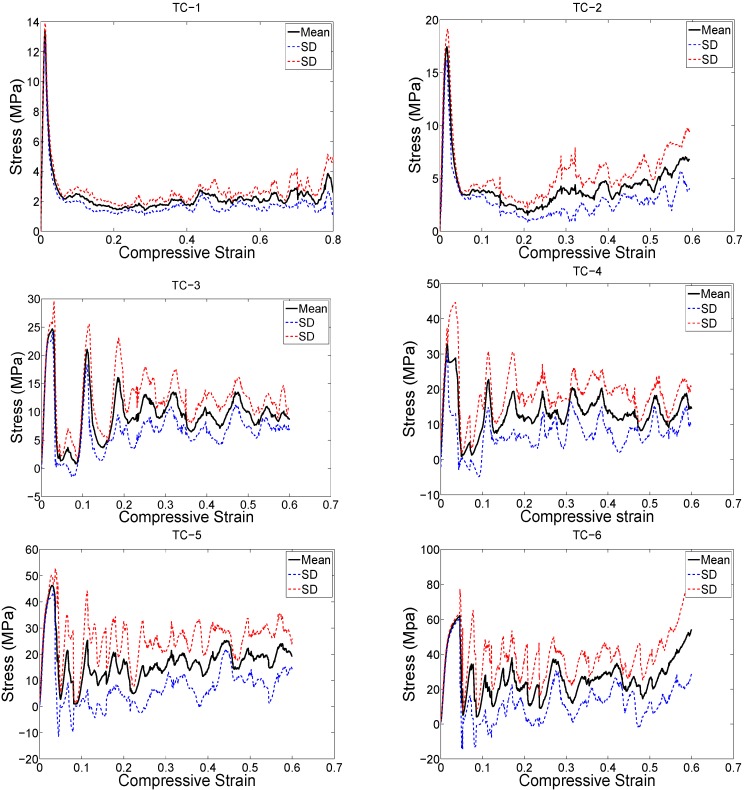
Compressive stress-*versus*-compressive strain curves for specimens based on the truncated cube unit cell and with different porosities (see Table 2).

**Figure 6:**
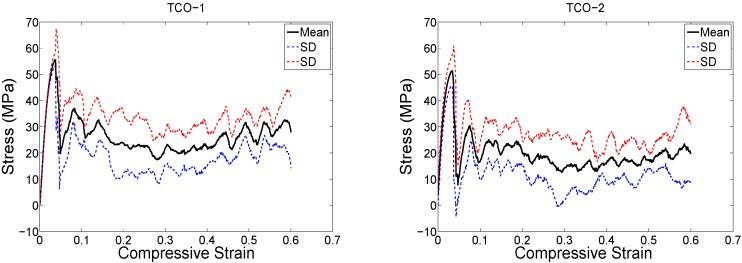

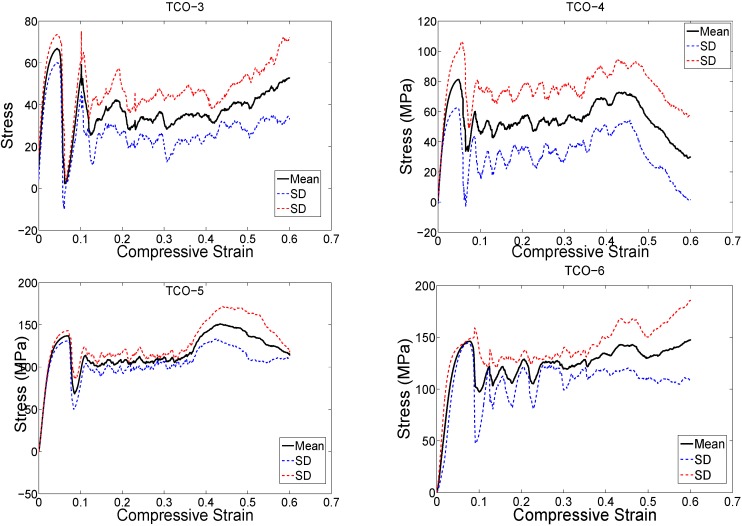
Compressive stress-*versus*-compressive strain curves for specimens based on the truncated cuboctahedron unit cell and with different porosities (see Table 2).

**Figure 7:**
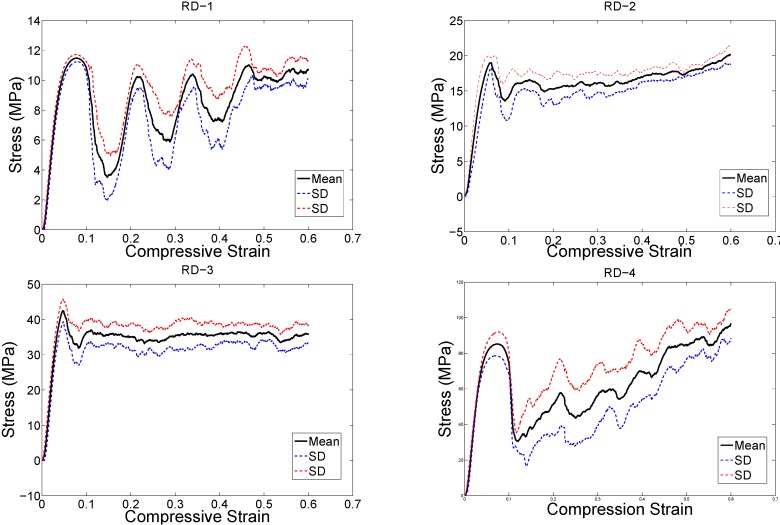

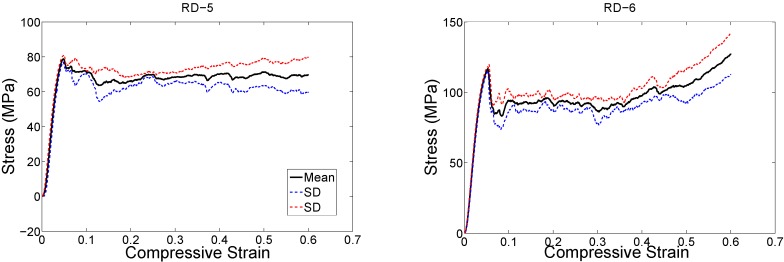
Compressive stress-*versus*-compressive strain for specimens based on the rhombic dodecahedron unit cell and with different porosities (see Table 2).

**Figure 8:**
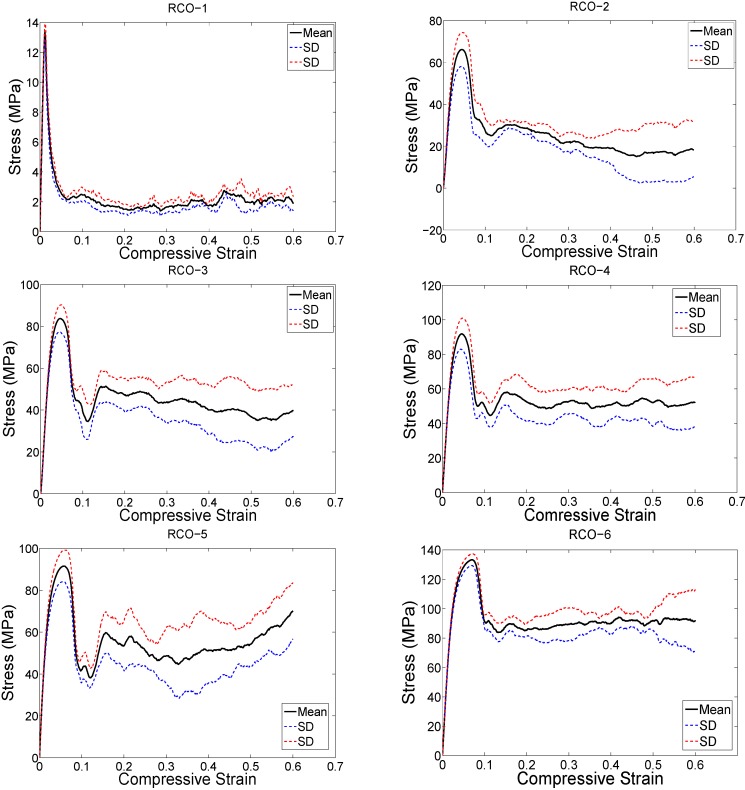
Compressive stress-*versus*-compressive strain curves for specimens based on the rhombicuboctahedron unit cell and with different porosities (see Table 2).

The structure relative density of each unit cell configuration is presented in Table 2. The trends observed in the stress strain curves of the specimens with different types of unit cells were quite different (Figure 3, Figure 4, Figure 5, Figure 6, Figure 7 and Figure 8). There were also differences in the shape of stress-strain curves of specimens with the same type of unit cell configuration but different relative density (RD) (Figure 3, Figure 4, Figure 5, Figure 6, Figure 7 and Figure 8). In many cases, the typical stress-strain response of porous alloy was observed including the initial linear response that was followed by a plateau region and the subsequent fluctuations of the stress-strain curve (Figure 3, Figure 4, Figure 5, Figure 6, Figure 7 and Figure 8). The final part of the stress-strain curves was often associated with stiffening of the porous structure (Figure 3, Figure 4, Figure 5, Figure 6, Figure 7 and Figure 8). In general, the level of fluctuations following the plateau region tended to decrease as the structure relative density of the porous structures increased (Figure 3, Figure 4, Figure 5, Figure 6, Figure 7 and Figure 8). However, this was not, the case for porous structures based on the truncated cube unit cell (Figure 8).

**Table 2:** Summary of the structure relative density results (in %).

	Structure Relative Density (%)			
	CAD File	Dry Weighing (SD)	Archimedes (SD)	μCT
**Cubic (C)**				
C-1	10	11 (0.1)	12 (0.1)	13
C-2	22	21 (0.2)	22 (0.2)	24
C-3	27	26 (0.2)	26 (0.2)	28
C-4	35	34 (0.1)	34 (0.2)	37
**Diamond (D)**				
D-1	11	11 (0.1)	11 (0.2)	11
D-2	21	20 (0.2)	21 (0.1)	21
D-3	28	26 (0.4)	27 (0.3)	28
D-4	37	34 (0.3)	35 (0.4)	36
**Truncated cube (TC)**				
TC-1	6	7 (0.1)	7(0.1)	9
TC-2	9	9 (0.1)	9 (0.1)	11
TC-3	12	12 (0.1)	12 (0.1)	12
TC-4	16	14 (0.2)	15 (0.2)	14
TC-5	21	17 (0.2)	18 (0.1)	17
TC-6	24	20 (0.2)	20 (0.2)	20
**Truncated Cubeoctahedron (TCO)**				
TCO-1	18	20 (0.4)	20 (0.4)	19
TCO-2	21	23 (0.2)	23 (0.2)	21
TCO-3	26	25 (0.5)	25 (0.5)	23
TCO-4	31	28 (0.2)	28 (0.3)	28
TCO-5	34	31 (0.3)	31 (0.3)	32
TCO-6	36	34 (0.2)	35 (0.3)	36
**Rhombicdodecahdron (RD)**				
RD-1	10	11 (0.3)	11 (0.4)	11
RD-2	15	17 (0.2)	17 (0.1)	16
RD-3	20	23 (0.2)	23 (0.1)	22
RD-4	25	27 (0.1)	27 (0.2)	27
RD-5	29	28 (0.3)	28 (0.3)	28
RD-6	34	33 (0.3)	33 (0.2)	32
**Rhombic Cubeoctahedron (RCO)**				
RCO-1	16	18 (0.2)	18 (0.2)	18
RCO-2	18	21 (0.2)	21 (0.2)	21
RCO-3	21	23 (0.3)	23 (0.3)	24
RCO-4	26	25 (0.3)	26 (0.4)	25
RCO-5	31	29 (0.4)	29 (0.4)	27
RCO-6	36	32 (0.3)	33 (0.5)	31

**Figure 9:**
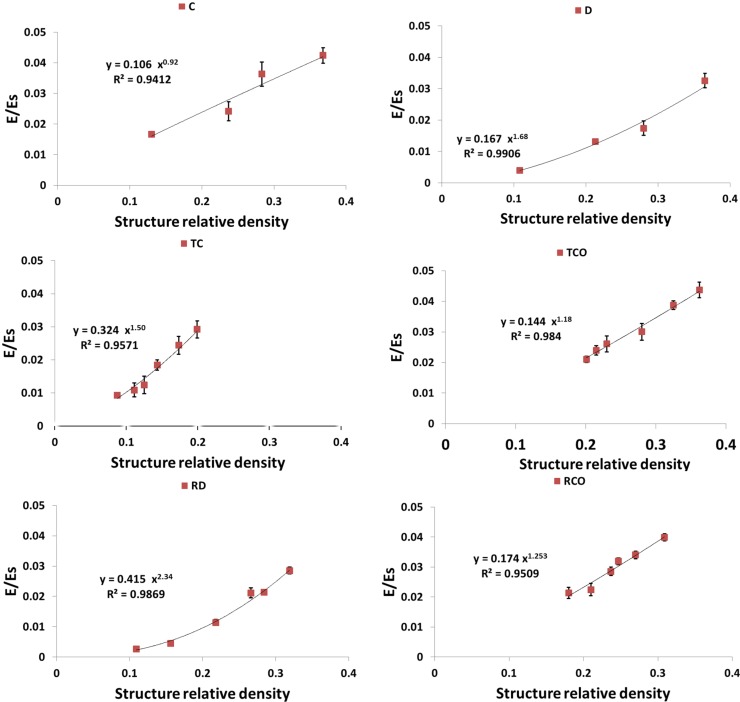
Summary of the elastic gradient results for porous structures basedon different types of unit cell configurations (cubic (C); diamond (D); truncatedcube (TC); truncated cuboctahedron (TCO); rhombic dodecahedron (RD); rhombicuboctahedron (RCO)) and different structure relative densities (see Table 2) (E_s_ indicates the elastic gradient of the structure if it was solid).

**Figure 10:**
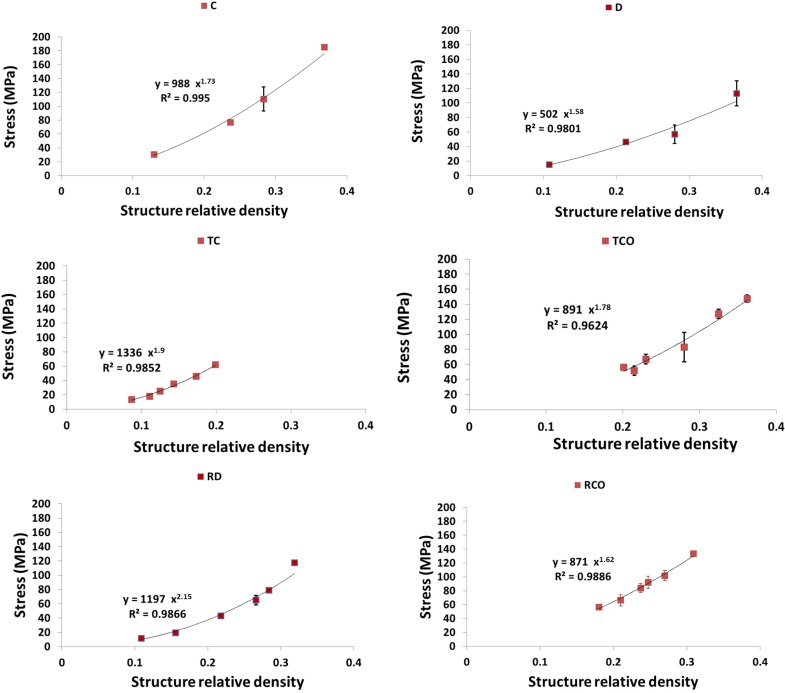
Summary of the first maximum stress results for porous structures based on different types of unit cell configurations (cubic (C); diamond (D); truncated cube (TC); truncated cuboctahedron (TCO); rhombic dodecahedron (RD); rhombicuboctahedron (RCO)) and different structure relative densities (see Table 2).

**Figure 11:**
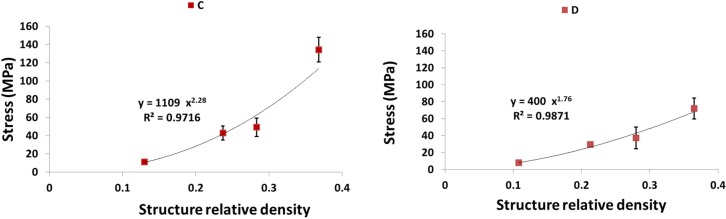

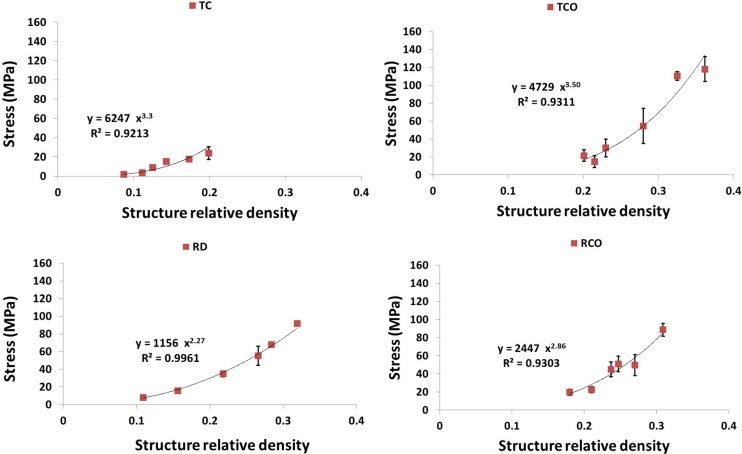
Summary of the plateau stress results for porous structures based on different types of unit cell configurations (cubic (C); diamond (D); truncated cube (TC); truncated cuboctahedron (TCO); rhombic dodecahedron (RD); rhombicuboctahedron (RCO)) and different structure relative densities (see Table 2).

**Figure 12:**
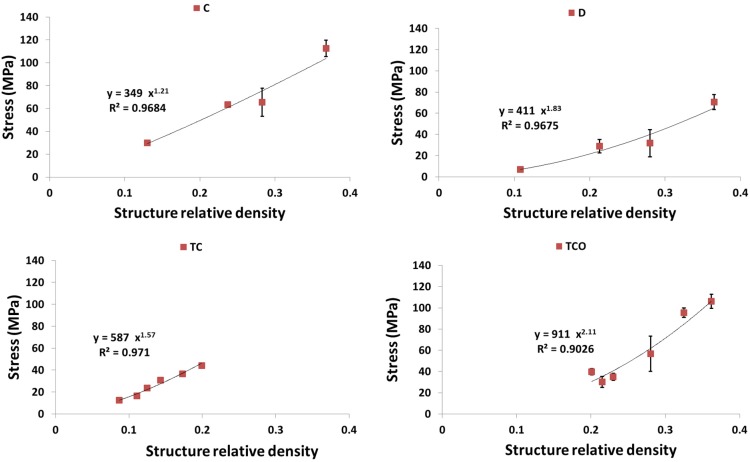

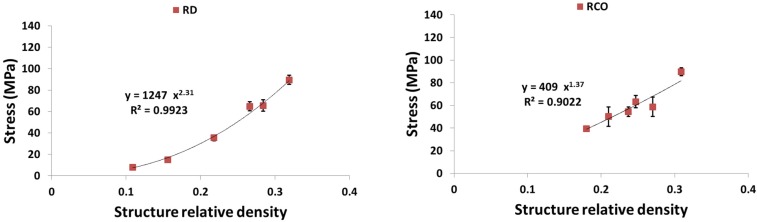
Summary of the yield stress results for porous structures based on different types of unit cell configurations (cubic (C); diamond (D); truncated cube (TC); truncated cuboctahedron (TCO); rhombic dodecahedron (RD); rhombicuboctahedron (RCO)) and different structure relative densities (see Table 2).

**Figure 13:**
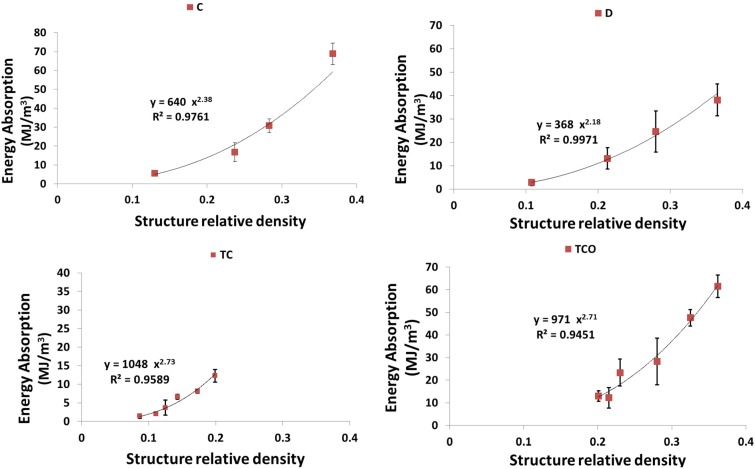

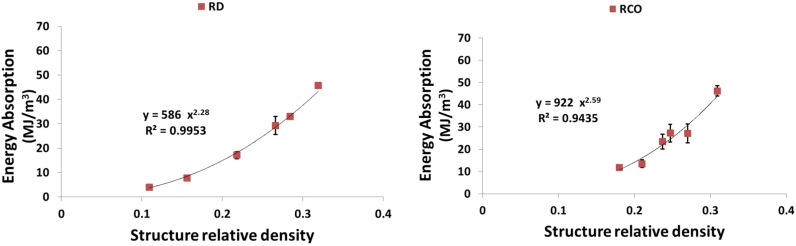
Summary of the energy absorption results for porous structures based on different types of unit cell configurations (cubic (C); diamond (D); truncated cube (TC); truncated cuboctahedron (TCO); rhombic dodecahedron (RD); rhombicuboctahedron (RCO)) and different structure relative densities (see Table 2).

As expected, each of the compressive properties increased with increase in structure relative density (Figure 9, Figure 10, Figure 11, Figure 12 and Figure 13). The exponent of the power law fitted to the experimental data points (Figure 9, Figure 10, Figure 11, Figure 12 and Figure 13) varied between 0.93 and 2.34 for the elastic gradient (Figure 9), between 1.28 and 2.15 for the first maximum stress (Figure 10), between 1.75 and 3.5 for the plateau stress (Figure 11), between 1.21 and 2.31 for the yield stress (Figure 12), and between 2.18 and 73 for energy absorption (Figure 13).

**Figure 14:**
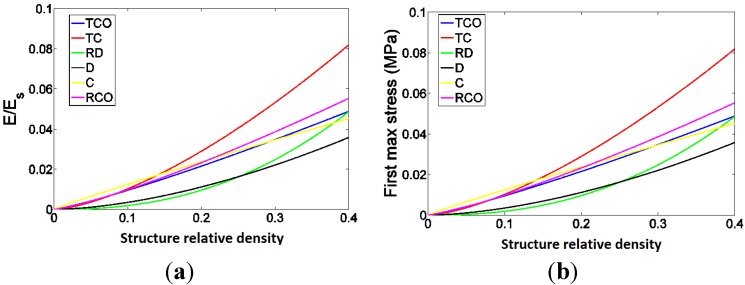

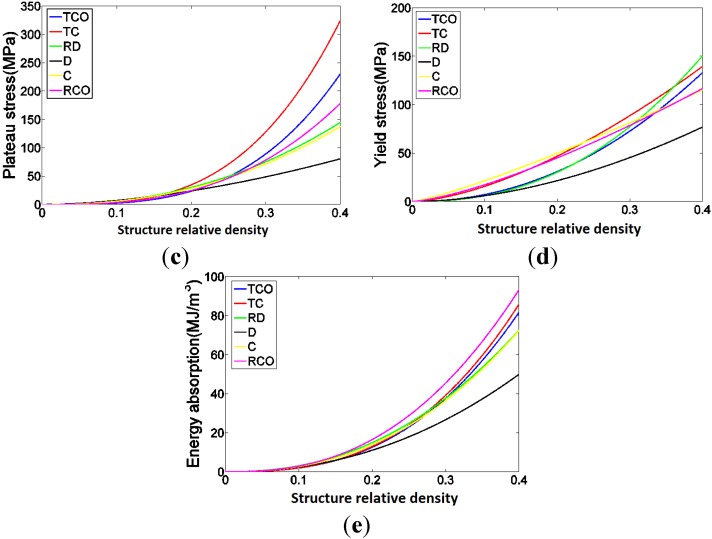
Comparison between the mechanical properties measured for different types of porous structures based on the six different unit cells studied here including (**a**) Elastic gradient; (**b**) First maximum stress. (**c**) Plateau stress; (**d**) Yield stress; (**e**) Energy absorption. In these figures, the power laws fitted to the experimental data points, and not the experimental data points themselves, are compared with each other.

Among all the unit cells studied here, the structure with the diamond unit cell was the most compliant, especially at RD > 0.15, whereas the stiffest structure was that having a truncated cube unit cell, especially when RD > 0.30 (Figure 9). When RD was small (RD < 0.2) the structures may be divided into two groups, with those in the first group (truncated cube, truncated cuboctahedron, rhombicuboctahedron, and cube unit cells) having larger stiffness than those in the second group (diamond and rhombic dodecahedron unit cells) (Figure 9 and Figure 14a).

With regard to σmax, there is also separation of the structures into two groups. When RD < 0.2, the structures with the highest and lowest value of this compressive property were built using rhombiccuboctahedron and rhombic dodecahedron unit cells, respectively (Figure 10). However, when RD > 0.2, the structures with the highest and lowest value of this compressive property were built using the truncated cube and diamond unit cells, respectively (Figure 10 and Figure 14b). When RD < 0.2, there is no difference in plateau stress between the different structures, but, when RD > 0.2, the highest and lowest value of this compressive property were built using the truncated cube and diamond unit cells, respectively (Figure 11 and Figure 14c). The four remaining unit cells are relatively close in terms of the plateau stress values they exhibit (Figure 11 and Figure 14c).

Regarding σy, structures with the diamond unit cell show the lowest value throughout the RD range (Figure 12 and Figure 14d). The one group comprising structures having the truncated cube rhombicuboctahedron, and cube and cube and the other group comprising structures having truncated cuboctahedron and rhombic dodecahedron, When RD < 0.2, the former group has clearly higher yield stress values as compared to the latter group, but, when RD > 0.2, the results for the two groups overlapped (Figure 12 and Figure 14d). When RD < 0.2, Energy absorption (EA) for the structures with different unit cell configurations are practically the same, but, at higher RD, EA of structure with diamond unit cell is much lower than that of a structure with any other type of unit cell configuration (Figure 13 and Figure 14e).

**Figure 15:**
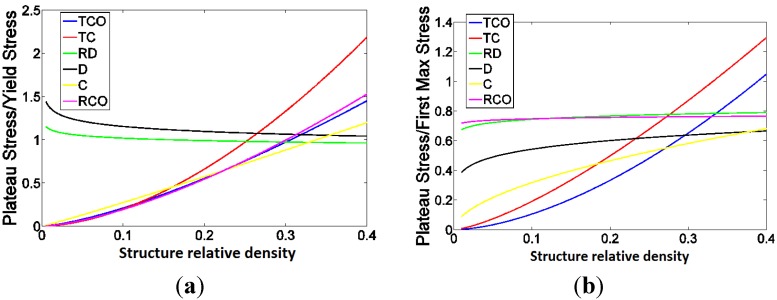
(**a**) The ratio of plateau stress to yield stress as well as (**b**) the ratio of plateau stress to first maximum stress for different types of unit cells. In these figures, the power laws fitted to the experimental data points, and not the experimental data points themselves, are compared with each other.

The ratio of plateau stress to yield stress was more or less constant and close to one for the diamond and rhombic dodecahedron unit cells (Figure 15a). For the other types of unit cells, the ratio of plateau stress to yield stress remarkably increased with the relative density (Figure 15a). As for the ratio of plateau stress to first maximum stress, it was relatively stable for diamond, rhombic dodecahedron, and rhombicuboctahedron (Figure 15b). For the three remaining types of unit cells, the ratio of plateau stress to first maximum stress drastically increased with the relative density Figure 15b).

## 4. Discussion

The results of this study clearly show the difference between the porous structures made using different types of unit cells. Not only do the mechanical properties of the porous structures differ drastically between the various unit cells studied here, the deformation and failure mechanisms change as well particularly at the plateau region as well as in the succeeding regions of the stress-strain curves. These different failure mechanisms are reflected in the different shapes of stress-strain curves.

### 4.1. Comparison between the Different Types of Unit Cells

Since all other parameters are kept constant during the manufacturing of the specimens, the only factor that differentiates the different classes of porous structures from each other is the geometry of unit cell. For example, it was observed that the unit cells that include vertical struts, exhibit a different failure mechanism as compared to the other unit cells. In the unit cells with vertical struts, failure of one (vertical) strut usually resulted in the collapse of the entire unit cell, causing a sudden drop of the measured force to values close to zero. Once one unit cell, that is often the weakest link in the remaining porous structure, has collapsed, the other unit cells take over the force-carrying function of the missing unit cell and the force increases again. This will continue until the next weakest link in the remaining porous structure has collapsed and the force drops to near-zero values again. The presence of vertical struts could not, however, explain all the cases where force repeatedly dropped to near-zero values. An important exception was the diamond unit cell. In this unit cell, the geometry of the unit cell is such that the failure of one strut could easily cause the collapse of the entire unit cell, as the shape of the unit cell is relatively simple and the different struts provide only limited support to each other. This could be also found back in all of the compressive properties measured for the diamond unit cell. Comparatively speaking, the diamond unit cell showed the lowest values of the compressive properties for the entire range of apparent densities. There are only two exceptions, elastic gradient and first maximum stress, where rhombic dodecahedron shows slightly lower compressive properties for the lowest values of the structure relative density.

The stiffness of the porous structures made from different types of unit cells is probably the most important property of these structures when they are used as bone-mimicking biomaterials. The elastic gradient is the best indicator of the stiffness of the porous structure, among all the compressive properties presented here. For small apparent densities, *i.e.*, < 0.15, one could speak of two groups of unit cells, namely strong unit cells and weak unit cells. The strong unit cells group includes truncated cube, truncated cuboctahedron, rhombicuboctahedron, and cube, while the weak unit cell group includes diamond and rhombic dodecahedron. Within each of the groups, there is not much difference between the different types of unit cells for small structure relative density values, meaning that they are interchangeable from mechanical viewpoint. The other considerations such as permeability [3,9] could therefore play more important role when deciding which of those unit cells is used in bone regeneration applications. For larger structure relative density values, *i.e.*, >0.15, the truncated cube unit cell shows remarkably higher stiffness values and could therefore be used in the applications where high stiffness values are required. Since cube and truncated cube are relatively similar unit cells, it is remarkable that such small variation in the geometry of the cubic unit cells results in such improvement in the stiffness values for relatively large apparent densities. One could explain this by noting that in the cube unit cell force transmission occurs at a few junction points that are also prone to stress concentration. Truncated cube replaces the single junction of the cubic unit cell with a supporting structure that could better distribute and transmit the forces. This improves the stiffness of the porous structure particularly for higher apparent densities where the thick struts at the truncation region of the truncated cube unit cell are particularly closed-pack and support the porous structure very efficiently.

### 4.2. Ratio of Plateau Stress to Yield Stress

One of the important findings of the current study is the point that the relationship between the plateau stress and yield stress is very different for different types of unit cells. In general, plateau stress has received more attention in the recent literature, partly because of the emphasis and explicit definition of the concept in the new ISO standard for the mechanical testing of metallic porous materials [51]. In comparison, there is less emphasis on the concept of yield (or compressive offset) stress in the standard, demoting it to the status of “optional information” in the standard test report [51]. As a consequence, a number of recent studies including our studies on porous structures made from the rhombic dodecahedron unit cell [16,40] and one study of the mechanical behavior of porous structures based on the diamond unit cell [10] have used the concept of plateau stress as a replacement for the yield stress. The results of the current study show that, interestingly, for both types of unit cells used in our previous studies, the plateau and yield stress are very close. Moreover, the ratio of plateau stress to yield stress is largely independent from the structure relative density. This justifies the use of plateau stress as a replacement for the yield stress for the porous structures based on those two types of unit cells. The results of this study, however, show that this is not necessarily the case for the other types of unit cells. Not only the plateau and yield stress are not close to each other for the other types of unit cells, their ratio could be very much dependent on the structure relative density. This is an important point in all future studies where one needs to choose a specific parameter for representing the elastic limit of additively manufactured porous structures based on the different types of unit cells.

### 4.3. Energy Absorption

Fracture toughness of bone is defined as the resistance to crack growth before the final fracture [54] and several studies on what can influence on fracture toughness of the human bone, cortical and trabecular [55,56,57,58] show the importance of this definition. Although tough bone resists more to fracture but it may have lower yield point and be considered weaker [59]. It is therefore important to select the right type of unit cell for bone-mimicking porous structure by comparing the energy absorption values of the porous structures based on the different types of unit cells with that of bone they are aimed to replace. This is an important design aspect has received less attention in the previous studies that look into the mechanical properties of bone-mimicking porous biomaterials and how they are related to those of bone.

### 4.4. Anisotropy

The mechanical properties of porous structures based on some of the unit cells included in the current study are anisotropic. In the current study, we only studied the mechanical properties of the porous structures in one direction (Figure 1). The mechanical properties of the porous structures may be therefore very different in the directions not tested in the current study. One needs to be careful when interpreting the results presented here, as they only pertain to specific directions of unit cells. The experiment required for characterizing the mechanical properties of the porous structures in all relevant directions is formidably large and expensive. A more feasible approach would be to develop analytical and computational models that are first validated against the experimental data presented here and could then be used for estimating the mechanical properties of the porous structures in all possible directions. In addition to the anisotropy caused by the geometry of the unit cells, the manufacturing process could also cause some directionality in the porous structure [44]. This directionality, which is dependent on the geometry of the unit cell, could also induce some additional anisotropy in the mechanical behaviour of the porous structures.

### 4.5. Applications in the Design of Implants and Tissue Engineering Scaffolds

The main application of the results presented in the current study is in the design of porous biomaterials used for bone substitution either as an implant or as a part of a bone tissue engineering scheme. The mechanical properties of the porous biomaterials are important from several viewpoints. First, one needs to ensure that there is a good match between the stiffness of porous biomaterial and those of the bone they replace. This could help in avoiding stress shielding. The elastic gradient values reported here for the different types of unit cells could be important in that context. Second, it is important to make sure that the porous biomaterials are capable of providing enough mechanical support and do not fail under the mechanical loading they are exposed to. The plateau stress as well as yield and first maximum stress values reported here could play important roles in that regard.

From a design viewpoint, one needs to ensure that the mechanical properties of the porous biomaterials are favorable for bone regeneration and ingrowth. That is because bone tissue formation is known to be largely driven by mechanical loading [60,61,62,63]. The results of the current study clearly show that, for the same structure relative density, the mechanical properties of bone-mimicking porous biomaterials are very much dependent on the morphology of the porous structure including the type of unit cell and the unit cell dimensions. On the other hand, the same morphological properties determine the other important properties of the porous biomaterials such as permeability and diffusivity [2,3,8,9]. The design of porous biomaterials for bone regeneration applications can therefore be defined as a multi-objective optimization problem. There are additional patient-specific aspects that need to be taken into account. It is therefore important to combine the computer models for optimal design of porous biomaterials with patient-specific finite element models of bones [64,65,66]. The complex and multi-objective nature of such an optimal design problem requires a high degree of flexibility in the design space. Studies such as the present study that help to establish the relationship between the morphological design and the different types of properties of porous biomaterials based on various types of unit cells are helpful in this context. That is because they enable the designers to use a larger library of unit cells for which the different types of properties including mechanical properties are known, thereby enlarging the design space for optimal design of bone substituting implants and tissue engineering scaffolds. Given the production flexibility offered by advanced additive manufacturing techniques such as selective laser melting, different types of unit cells could be combined in one single implant or scaffold so as to optimally distribute the properties within the entire implant or scaffold.

The results presented in this study are also valuable for corroboration of analytical and numerical models that are developed used for prediction of the mechanical properties of porous structures given their designed morphology. This type of experimental data is not currently available in the literature particularly for some of the unit cells studied here.

### 4.6. Future Research

In this study, all the manufacturing parameters such as building orientation and post processing of the samples [44] or laser power or energy density of the specimens processed by SLM [30] considered to be constant. Changing in any of these parameters will influence the results [42]. It is clear from the results of this study that the deformation and failure mechanisms of porous structures based on the considered unit cells are very different. Even though certain aspects of the deformation and failure mechanisms were studied in the current study, it was not the main focus of the paper. It is suggested that future studies should focus on the detailed deformation and failure mechanisms of additively manufactured porous biomaterials based on different types of unit cells. In particular, it would be useful to perform full-field strain measurement [67,68,69,70] during the mechanical testing of the structures, for example, using optical techniques such as digital image correlation (DIC). DIC has been previously used for measurement of strain in engineering [71,72,73,74] and biological materials [75,76,77] and is shown to be capable of capturing the detailed deformation and fracture mechanisms of both types of materials. For determining the mechanical properties only static compressive properties were determined in the present work. In future studies, other relevant mechanical properties, such as static bending strength [46], static torsional strength [46] and fatigue life [50], should be determined.

## 5. Conclusions

The relationship between morphological and mechanical properties of selective laser melted porous titanium alloy biomaterials based on six different types of space-filling unit cells were studied. It was observed that the mechanical behavior, mechanical properties, and failure mechanisms of the porous structures are highly dependent on the type and dimensions of the unit cells out of which the porous structures are made. As expected, compressive properties of all the porous structures increased with structure relative density. Moreover, for a given compressive property of a porous structure, the dependence on the structure relative density was of the power type. The exponent could be used for generalizing the relationships between structure relative density and the compressive properties of porous structures with different types of unit cells. When comparing the compressive properties of the porous structures based on the different types of unit cells, it was found that in many cases the comparative performance of the structures is different for low and high values of structure relative density with a separating structure relative density of 0.15–0.2. Among all unit cells, the diamond unit cell consistently showed lower compressive properties. Regarding the stiffness values, the unit cells were divided into a high stiffness group including truncated cube, truncated cuboctahedron, rhombicuboctahedron, and cube and a low stiffness group including diamond and rhombic dodecahedron. However, truncated cube showed remarkably higher stiffness than other members of its group for apparent densities exceeding 0.2. The results obtained in the present study revealed the relationship between the morphological and compressive properties of porous structures based on six different types of unit cells, many of which have been so far largely unexplored. Moreover, it could serves as a basis for validation of analytical and computational models developed for estimation of the mechanical properties of additively manufactured porous biomaterials.
